# Anticholinesterase Activity of Budmunchiamine Alkaloids Revealed by Comparative Chemical Profiling of Two *Albizia* spp., Molecular Docking and Dynamic Studies

**DOI:** 10.3390/plants11233286

**Published:** 2022-11-29

**Authors:** Mai E. Hussein, Osama G. Mohamed, Ahlam M. El-Fishawy, Hesham I. El-Askary, Ahmed A. Hamed, Marwa M. Abdel-Aziz, Radwan Alnajjar, Amany Belal, Ahmed M. Naglah, Abdulrahman A. Almehizia, Ahmed A. Al-Karmalawy, Ashootosh Tripathi, Amira S. El Senousy

**Affiliations:** 1 Pharmacognosy Department, Faculty of Pharmacy, Cairo University, Kasr el Aini St., Cairo 11562, Egypt; 2 Natural Products Discovery Core, Life Sciences Institute, University of Michigan, Ann Arbor, MI 48109, USA; 3 Microbial Chemistry Department, National Research Centre, 33 El-Buhouth Street, Dokki, Giza 12622, Egypt; 4 Regional Center for Mycology and Biotechnology (RCMB), Al-Azhar University, Cairo 11651, Egypt; 5 Department of Chemistry, Faculty of Science, University of Benghazi, Benghazi 16063, Libya; 6 PharmD, Faculty of Pharmacy, Libyan International Medical University, Benghazi 16063, Libya; 7 Department of Chemistry, University of Cape Town, Rondebosch 7701, South Africa; 8 Medicinal Chemistry Department, Faculty of Pharmacy, Beni-Suef University, Beni-Suef 62514, Egypt; 9 Drug Exploration and Development Chair (DEDC), Department of Pharmaceutical Chemistry, College of Pharmacy, King Saud University, Riyadh 11451, Saudi Arabia; 10 Peptide Chemistry Department, National Research Centre, Dokki, Cairo 12622, Egypt; 11 Pharmaceutical Chemistry Department, Faculty of Pharmacy, Ahram Canadian University, 6th of October City, Giza 12566, Egypt; 12 Department of Medicinal Chemistry, College of Pharmacy, University of Michigan, Ann Arbor, MI 48109, USA

**Keywords:** anticholinesterase, *A. lucidior*, *A. procera*, budmunchiamine, UHPLC–QTOF, molecular networking, molecular docking, MM-GBSA

## Abstract

Alzheimer’s disease remains a global health challenge and an unmet need requiring innovative approaches to discover new drugs. The current study aimed to investigate the inhibitory activity of *Albizia lucidior* and *Albizia procera* leaves against acetylcholinesterase enzyme in vitro and explore their chemical compositions. Metabolic profiling of the bioactive plant, *A. lucidior*, via UHPLC/MS/MS-based Molecular Networking highlighted the richness of its ethanolic extract with budmunchiamine alkaloids, fourteen budmunchiamine alkaloids as well as four new putative ones were tentatively identified for the first time in *A. lucidior*. Pursuing these alkaloids in the fractions of *A. lucidior* extract via molecular networking revealed that alkaloids were mainly concentrated in the ethyl acetate fraction. In agreement, the alkaloid-rich fraction showed the most promising anticholinesterase activity (IC_50_ 5.26 µg/mL) versus the ethanolic extract and ethyl acetate fraction of *A. lucidior* (IC_50_ 24.89 and 6.90 µg/mL, respectively), compared to donepezil (IC_50_ 3.90 µg/mL). Furthermore, deep in silico studies of tentatively identified alkaloids of *A. lucidior* were performed. Notably, normethyl budmunchiamine K revealed superior stability and receptor binding affinity compared to the two used references: donepezil and the co-crystallized inhibitor (MF2 700). This was concluded based on molecular docking, molecular dynamics simulations and molecular mechanics generalized born/solvent accessibility (MM–GBSA) calculations.

## 1. Introduction

Alzheimer’s disease (AD) is the most frequent form of dementia and the seventh major cause of mortality worldwide with more than 55 million people suffering from dementia, predicted to reach 139 million by 2050 [1]. AD is a degenerative brain disorder, clinically manifested by a disturbance in memory and judgment ability, personality changes, agitation and sleep abnormalities [1,2]. It represents a global health challenge, becoming a crucial socioeconomic burden with the increasing life expectancy and decreasing mortality rates [1]. The use of acetylcholinesterase inhibitors (AChEIs) as a group of medications for AD treatment has been crucial in ameliorating cognitive performance [3]. The adverse effects reported for donepezil, tacrine, galantamine and rivastigmine, FDA-approved AChEIs drugs for AD treatment, include gastrointestinal disorders such as nausea, anorexia, diarrhea, abdominal pain and an increase in cardiac vagal tone causing bradycardia [2,3]. Therefore, the development of safe, more effective drugs is of utmost importance.

In that context, natural plants have been a valuable source for discovering new cholinesterase inhibitors for the treatment of AD such as *Withania somnifera, Convolvulus pluricaulis* and *Centella asiatica* [4,5]. The conventional bioassay-based methods for discovering novel bioactive natural compounds are oftentimes intensive with high rates of re-isolation of known structures [6]. The richness of plants with a plethora of bioactive metabolites motivated scientists to explore them with interesting chemistries. Consequently, new strategies for dereplication processes, involving mass spectrometry integrated with wide online databases such as PubChem, ChemSpider, and Global Natural Product Social Molecular Networking (GNPS), have attracted more attention to allow the rapid identification of bioactive metabolites in a complex extract [7,8]. Molecular Networking (MN) via GNPS has emerged as a new approach enabling metabolite annotation together with featuring discriminating components [6,9]. It is a computational technique that contributes to the interpretation of complex data from MS analysis, identifying the similarities among all MS/MS spectra and propagating annotation to unknown but related molecules [10]. On the other hand, molecular docking is a widely known and applied computational tool to shorten the time span of the drug discovery process for treating different chronic diseases, as it assists researchers to propose and/or study the current mode of action for a particular drug member [11].

The genus *Albizia*, of the family Fabaceae, is a rich source of flavonoids, alkaloids, terpenes and saponins, etc. [12]. *Albizia* species are used in folk medicine to treat various ailments such as rheumatism, diarrhea, cough, wounds and stomachache, in addition to their traditional use in treating CNS disorders such as anxiety, depression, and AD in Southern Africa [12,13,14]. The efficacy of some *Albizia* species against AD has been previously reported [13,15,16,17,18,19,20]. However, the effectiveness of *Albizia lucidior* (Steud.) I.C.Nielsen (commonly known as the potka siris) and *Albizia procera* (Roxb.) Benth. (commonly known as the white siris) leaves in the treatment of AD has not been explored yet. Additionally, only few studies were found about the chemical potential of *A. procera* leaves and aerial parts [21,22,23,24,25,26,27], whereas nothing was traced regarding *A. lucidior* leaves. Therefore, an in-depth exploration of their biological activity would definitely contribute towards the identification of drug leads for controlling AD. To note, our research group has previously investigated the antibacterial and cytotoxic effects of metabolites in the endophytic fungus *Aspergillus fumigatus*, isolated from *A. lucidior* leaves [28,29].

On that account, the current work focused on investigating the AChE inhibitory activities of *A. lucidior* and *A. procera* leaves and exploring their phytochemical profiles via applying UHPLC-ESI-QTOF-MS/MS-based molecular networking to distinguish the variation in their chemical constituents, highlighting metabolites contributing to bioactivity. Metabolites identified in the more promising species were further inspected by molecular docking to point out compounds with higher affinity for AChE than donepezil, the standard drug for AD treatment. Subsequently, molecular dynamics simulations of the most favorable candidates were carried out to examine the exact stability of the formed complexes and apprehend the contribution of specific amino acids with the inhibitors at the active site all over the simulation time.

## 2. Results and Discussion

### 2.1. Acetylcholinesterase Inhibitory Activities of A. lucidior and A. procera Leaves

**Figure 1:**
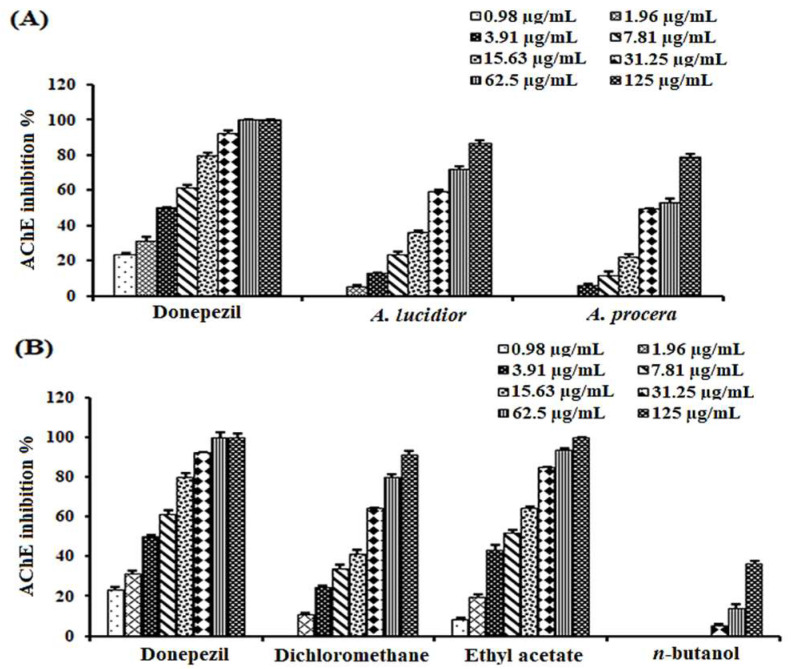
Acetylcholinesterase inhibitory activities of: (**A**) ethanolic extracts of *A. lucidior* and *A. procera* and (**B**) fractions of *A. lucidior*, compared to donepezil. Values are expressed as mean ± S.D.

The inhibitory activities of the ethanolic extracts of both species were tested towards AChE, revealing a reduction in the enzyme activity in a dose-dependent manner, comparable to donepezil as a positive standard (Figure 1A). *A. lucidior* extract retained ≈ 1.7 times higher inhibitory potency (IC_50_ = 24.89 ± 1.60 µg/mL) than that of *A. procera* (IC_50_ = 43.50 ± 2.10 µg/mL), compared to donepezil (IC_50_ = 3.90 ± 0.72 µg/mL) (Table 1). The AChE inhibitory activity of *A. procera* bark extract had been previously reported (40.71 ± 0.46% at 0.1 mg/mL) [15]. However, this is the first evidence of the anticholinesterase activities of the leaves of both tested species. This inspired us to dig deeper to investigate the activities of different fractions, obtained from the more potent species, *A. lucidior*. Testing the AChE inhibitory activity of different fractions of *A. lucidior*, the fractions inhibited AChE activity in the same manner as their mother extract (Figure 1B) with the ethyl acetate fraction being the most active one (IC_50_ value of 6.90 ± 0.96 µg/mL), compared to donepezil (IC_50_ = 3.90 ± 0.72 µg/mL) (Table 1).

### 2.2. Total Phenolic and Flavonoid Contents of A. lucidior and A. procera Leaves

The bioactivity differences observed between the tested ethanolic extracts led us to conduct a phytochemical composition study to help in the identification of potential metabolites responsible for the AChE inhibitory activity. Based on the relationship between plants’ anticholinesterase activities and their phenolic contents [30], it was necessary to estimate the total phenolic (TPC) and flavonoid (TFC) contents in both species, as well the most bioactive fraction (ethyl acetate fraction of *A. lucidior*). TPC of the ethanolic extracts of *A. lucidior* and *A. procera* leaves were slightly similar (174.14 ± 3.20 vs. 176.01 ± 2.92 mg GAE/g extract, respectively), whereas TFC of *A. procera* leaves was marginally higher than that of *A. lucidior* (reaching 101.90 ± 5.57 vs. 86.26 ± 5.00 mg rutin/g extract, respectively). Interestingly, the ethyl acetate fraction of *A. lucidior* comprised only about 37.6 and 68.7% of the TPC and TFC of its mother ethanolic extract, respectively (65.43 ± 2.88 mg GAE/g fraction vs. 59.29 ± 2.94 mg rutin/g fraction). The results clearly suggested that the AChE inhibitory activity may not correlate with the extracts’ phenolics content. Therefore, further investigation to identify other phytochemicals that may play a key role is warranted.

### 2.3. Dereplication of Metabolites via UHPLC-ESI-QTOF-MS/MS-Based Molecular Networking

Metabolite profiling of both species under investigation was carried out via UHPLC-ESI-QTOF-MS/MS-based molecular networking, in an attempt to identify other potential metabolites with intended bioactivity. Metabolites were tentatively annotated based on their retention times, molecular formulae and their fragmentation patterns, compared to earlier reported data aided with GNPS spectral library search and Sirius. As listed in Table 2, 118 metabolites were identified, belonging to different classes including sterols, terpenes, fatty acids, sphingolipids, coumarins, phenolic acids, flavonoids, saponins and alkaloids. The results of identified metabolites in positive mode are shown in Table 2, while those of negative ionization mode are in supplementary data (Table S1), they are used as complementary data for the dereplication strategy. This study is the first to explore the detailed phytochemical comparative analysis of the current investigated *Albizia* species.

Visual analysis of MS/MS data via molecular networking enables annotation of metabolites, together with highlighting discriminating features between dissimilar samples [9], which are *A. lucidior* and *A. procera* in the current study. Therefore, further molecular networking, developed using GNPS system, was imported to Cytoscape 3.9.1 to visualize MS/MS data. Within the network, each node correlates to one consensus MS/MS spectrum, representing precursor ion mass (*m*/*z*). Nodes showing common fragmentation spectra are connected with edges. The node color denotes the sample’s origin (plant species or fraction herein), whereas the node size expresses the precursor ion intensity. These nodes were displayed as pie charts, reflecting the relative abundance of each ion in the tested samples. Two molecular networks were separately displayed for both species under investigation in positive (Figure S1) and negative (Figure S2) ionization modes.

**Figure 2:**
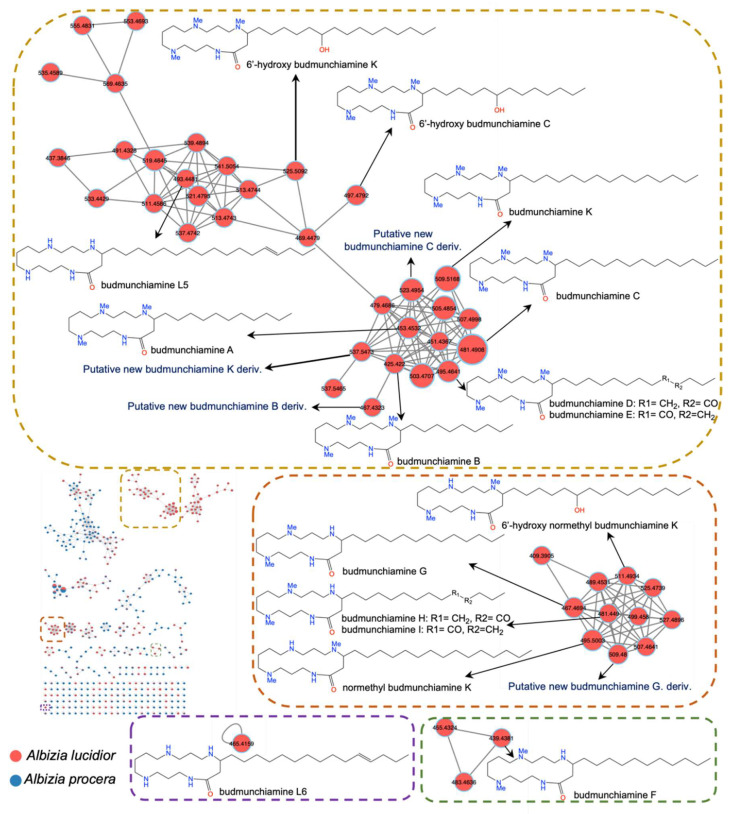
GNPS molecular network of two *Albizia* spp. ethanolic extracts in the positive ion mode. Each node is displayed as a pie chart representing relative abundance of the metabolite with red and blue colors in the ethanolic extracts of *A. lucidior* and *A. procera*, respectively. The node label represents precursor mass (*m*/*z*). The node size represents the sum of precursor ion intensity.

Interestingly, UHPLC-MS/MS based molecular networking in positive mode unearthed the abundance of macrocyclic spermine-based alkaloids, namely budmunchiamines previously isolated from other *Albizia* species [31,32,33,34,35], found exclusively in *A. lucidior* (Figure 2). The robust structure connectivity between the metabolites was noticed and supported by a high correlation cosine value of more than 0.90.

#### Identification of Budmunchiamine Alkaloids

Herein, we report the tentative identification of at least fourteen budmunchiamine alkaloids and four new putative alkaloids belonging to the budmunchiamine class for the first time in positive mode of *A. lucidior* only (Table 2 and Figure 2).

**Figure 3:**
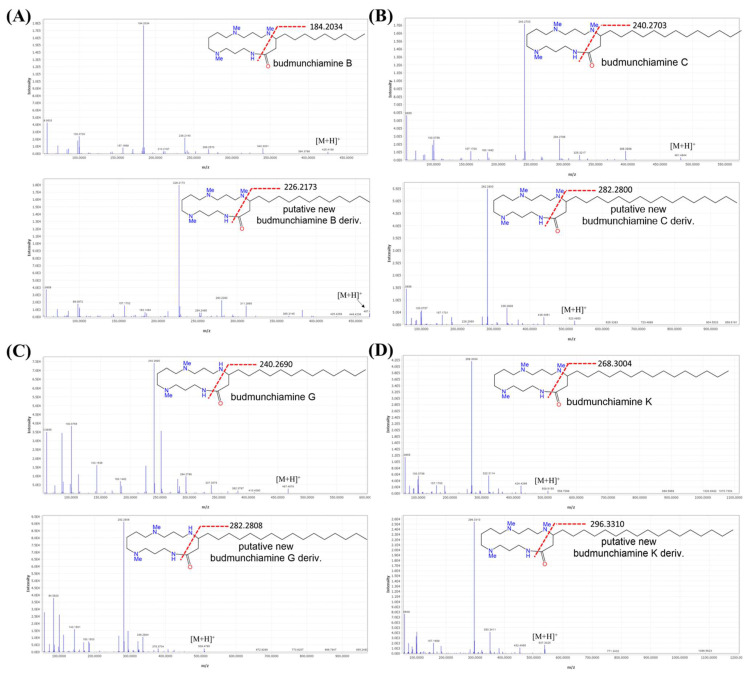
MS/MS fragmentation of budmunchiamine alkaloids and their putative derivatives: (**A**) budmunchiamine B; (**B**) budmunchiamine C; (**C**) budmunchiamine G and (**D**) budmunchiamine k, respectively.

Structurally, budmunchiamine alkaloids consist of a macrocyclic lactam ring as the basic skeleton, containing spermine moiety and an aliphatic chain at C4. They differ only in the length of the aliphatic chain and the substitution position of the methyl group on the macrocyclic ring (Figure 2). Mass fragmentation showed product ions at *m*/*z* 297, 283 and 255, representing macrocyclic rings containing three *N*-methyl groups, two *N*-methyl groups and devoid of any *N*-methyl groups, respectively. The MS data had been previously reported, showing the normal aliphatic chain fragmentation pattern with initial loss of a terminal methyl group followed by successive loss of fragments attributable to [Me(CH_2_)*n*]^+^ [33]. Moreover, at least three potential new budmunchiamine derivatives at 467.4323 *m*/*z* [M + H]^+^, *m*/*z* 523.4954 [M + H]^+^, *m*/*z* 509.4800 [M + H]^+^ with a mass difference of 42, representing a propene group, from budmunchiamine B at *m*/*z* 425.4220 [M + H]^+^, budmunchiamine C at *m*/*z* 481.4908 [M + H]^+^, budmunchiamine G at *m*/*z* 467.4694 [M + H]^+^, respectively, with a cosine score more than 0.95 were observed in our analysis (Figure 2). In addition, another new putative unknown compound was observed with *m*/*z* [M + H]^+^ value of 537.5473 (mass difference of 28), representing an ethylene group, from budmunchiamine K at *m*/*z* 509.5168 [M + H]^+^ with a cosine score 0.98. As shown in Figure 3, the putative new derivatives of budmunchiamine alkaloids showed nearly similar MS/MS fragmentation to the parent compounds with different base peaks. However, due to the limitation of MS-based characterization, several new alkaloids originating from clusters of the budmunchiamine class remained unannotated.

Budmunchiamine alkaloids exhibited promising health-promoting activities, such as antioxidant [36], anti-cancer [35], antimicrobial [35,36,37], and against Alzheimer’s disease [38]. Furthermore, the potential key role of alkaloids as anticholinesterase phytoconstituents had been previously reviewed [39]. Thus, we can speculate that the presence of budmunchiamine alkaloids in *A. lucidior* has been linked to its AChE inhibitory activity.

**Figure 4:**
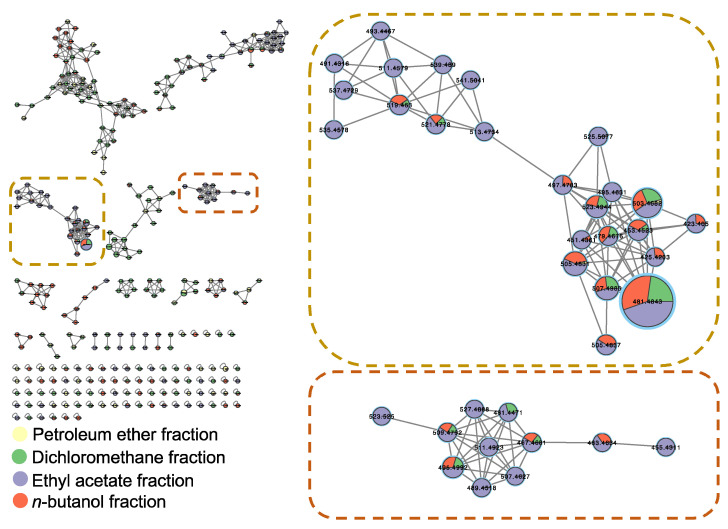
GNPS molecular network of different fractions of *A. lucidior* ethanolic extract in the positive ion mode. Each node is displayed as a pie chart representing the relative abundance of the metabolite with different colors in different fractions. The node label represents precursor mass (*m*/*z*). The node size represents the sum of precursor ion intensity.

In order to support our hypothesis, UHPLC-MS/MS-based molecular networking of different *A. lucidior* fractions *viz*., petroleum ether, dichloromethane, ethyl acetate and *n*-butanol was performed. Interestingly, on tracking the class of budmunchiamine alkaloids in the former fractions, the most potent fraction (ethyl acetate fraction) was found enriched with different budmunchiamine alkaloids, compared to other investigated fractions (Figure 4). These findings necessitated evaluating the anticholinesterase activity of the alkaloid-rich fraction of *A. lucidior* ethyl acetate extract.

**Table 2:** Identified metabolites in the positive ion mode of ethanolic extracts of *A. lucidior* and *A. procera* leaves using UHPLC-MS/MS.

No.	R_t_ (min)	Name	Ion *m*/*z* ppm	Molecular Formula	MS/MS Fragmentation Product Ions	Ref.	Al	Ap
1	0.40	Anthranilic acid	138.0551	C_7_H_8_NO_2_^+^	120.0349; 94.0651; 92.0497	[40]	√	-
2	1.62	Herniarin	177.0538	C_10_H_9_O_3_^+^	149.0575; 145.0266; 133.0982 117.0317	[41]	-	√
3	2.08	Budmunchiamine L6	465.4159	C_28_H_57_N_4_O^+^	380.3221; 278.2137; 266.1944 224.2000; 183.1474; 143.1170 100.0759; 86.0960; 58.0654	[34]	√	-
4	2.55	Budmunchiamine L5	493.4481	C_30_H_61_N_4_O^+^	408.3579; 294.2570; 288.2337 255.0398; 252.2334; 183.1498 157.1707; 100.0764;98.0973 84.0818; 72.0818; 58.0661	[34]	√	-
5	2.60	6′-Hydroxy budmunchiamine C	497.4792	C_29_H_61_N_4_O_2_^+^	412.3906; 310.2748; 256.2644 183.1497; 157.1703; 100.0759 98.0969; 86.0967; 72.0812 58.0658	[32]	√	-
6	2.60	Budmunchiamine B	425.4220	C_25_H_53_N_4_O^+^	340.3301; 295.7907; 269.2570 238.2140; 184.2034; 170.1875 157.1669; 100.0730; 98.0940 86.0938; 72.0785; 58.0633	[42]	√	-
7	2.63	Budmunchiamine-H/I	481.4490	C_28_H_57_N_4_O_2_^+^	351.3365; 308.2587; 282.2809 266.2484; 254.2483; 183.1493 143.1547; 112.1121; 100.0760 84.0815; 58.0660	[31]	√	-
8	2.67	Budmunchiamine D/E	495.4641	C_29_H_59_N_4_O_2_^+^	410.3745; 308.2592; 298.2726 254.2486; 183.1498; 157.1702 100.0760; 98.0969; 86.0968 72.0812; 58.0660	[31]	√	-
9	2.94	6′-Hydroxy-normethyl budmunchiamine K	511.4934	C_30_H_63_N_4_O_2_^+^	381.3848; 338.3056; 324.3253 284.2954; 283.2834; 183.1490 143.1542; 112.1119; 100.0755 84.0808; 72.0809; 58.0565	[32]	√	-
10	2.96	6′-Hydroxy budmunchiamine K	525.5092	C_31_H_65_N_4_O_2_^+^	440.4201; 338.3040; 297.2970 284.2934; 183.1471;157.1680 100.0738; 98.0947; 86.0945 72.0792; 58.0640	[33]	√	-
11	2.99	Budmunchiamine A	453.4532	C_27_H_57_N_4_O^+^	368.3624; 297.2891; 266.2470 212.2360 183.1475; 157.1683 100.0742; 98.0950; 86.0949 72.0796 58.06420	[42]	√	-
12	3.24	Budmunchiamine F	439.4381	C_26_H_55_N_4_O^+^	382.3778; 283.2806; 262.2502 227.2287: 212.2357; 185.1633 143.1524; 112.1106; 100.0740	[31]	√	-
13	3.28	Budmunchiamine G	467.4694	C_28_H_59_N_4_O^+^	337.3575; 294.2788 283.3109 252.2686; 240.2690; 183.1492 143.1536; 112.1117; 100.0755 84.0809; 72.0808; 58.0655	[33]	√	-
14	3.31	Budmunchiamine C	481.4908	C_29_H_61_N_4_O^+^	396.3958; 297.2891; 294.2795 240.2703; 183.1492; 157.1700 100.0756; 98.0963 86.0967 72.0809; 58.0655	[42]	√	-
15	3.69	Normethyl budmunchiamine K	495.5003	C_30_H_63_N_4_O^+^	322.3106; 283.3145; 268.3006 183.1488; 143.1538; 112.1120 100.0756; 84.0809; 72.0806 58.0655	[33]	√	-
16	3.90	Budmunchiamine K	509.5168	C_31_H_65_N_4_O^+^	424.4266; 322.3114 297.3351 268.3004; 183.1490; 157.1700 100.0756; 98.0965; 86.0964 72.0807; 58.0655	[33]	√	-
17	4.79	β-sitosterol	415.2124	C_29_H_51_O^+^	273.0765;135.0809;119.0864 107.0864	[43]	√	√
18	4.85	Stigmasterol	395.3678	C_29_H_47_^+^ [M-H_2_O + H]^+^	255.2623; 173.1322;159.1180 147.31178; 83.0863	[44]	√	-
19	4.97	Stearidonic acid	277.2166	C_18_H_29_O_2_^+^	259.2056; 235.1665; 149.1333 135.1173; 121.1012; 107.0858 93.0702	[45]	√	√
20	5.79	Arachidonic acid	305.2466	C_20_H_33_O_2_^+^	259.02036; 135.1159; 121.1000 107.0845; 93.0691; 55.0539	[45]	√	√
21	5.85	Linolenic acid ethyl ester	307.2260	C_20_H_35_O_2_^+^	261.2217; 243.2109;135.1185 123.1165; 109.1006; 95.0854 81.0701; 67.0544	[46]	√	√
22	6.18	3-*O*-[glucosyl (1⟶3)-glucoside]-28-*O*-[rhamnosyl (1⟶2) arabinoside] zanhic acid	1121.6241	C_53_H_85_O_25_^+^	959.5738; 843.3981; 519.2929	[47]	√	√
23	6.21	Pheophorbide B	607.2561	C_35_H_35_N_4_O_6_^+^	579.2601; 547.2351; 519.2391 505.2262; 475.2124; 447.2188 433.2024; 419.2213	[41]	√	√
24	6.48	Pheophorbide A	593.2781	C_35_H_37_N_4_O_5_^+^	533.2570; 505.2267; 461.2358 447.2196; 433.2369; 307.2628 177.1113; 133.0853	[41]	√	√
25	7.04	3-*O*-[arabinosyl(1→6)] -2-acetamido-2-deoxy-glucosyl oleanolic acid	792.5636	C_43_H_70_NO_12_^+^	457.3664; 336.2624; 335.2592	[48]	√	√
26	7.12	3-*O*-glucoside-28-*O*-[rhamnosyl-(1→2)-arabinoside] medicagenic acid	965.6190	C_47_H_74_O_19_Na	803.5684; 687.3933; 525.3411	[49]	√	√
27	7.78	Sapindoside B	905.5798	C_46_H_74_O_16_Na	627.2820; 495.2353	[50]	√	-
28	8.31	Julibroside JA_2_	911.6747	C_47_H_75_O_17_^+^	633.4474; 471.2672	[51]	√	√
29	8.37	Julibroside JA_3_	952.7286	C_49_H_78_NO_17_^+^	822.7053; 674.5065	[51]	√	-
30	8.48	3-*O*-[rhamnosyl (1→2)-arabinosyl(1→2)glucoside]-2-hydroxy oleanolic acid	913.6884	C_47_H_77_O_17_^+^	635.4620	[49]	√	-
31	8.93	Sapinoside A	773.5747	C_41_H_66_O_12_Na	495.2767	[50]	-	√

√, found; -, not found; Al, *A. lucidior*; Ap, *A. procera.*

### 2.4. Acetylcholinesterase Inhibitory Activity of Alkaloid-Rich Fraction of A. lucidior Ethyl Acetate Fraction

**Figure 5:**
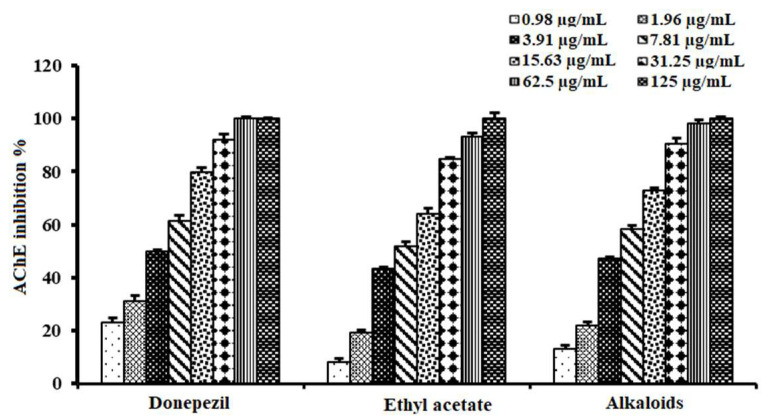
Acetylcholinesterase inhibitory activity of alkaloids from ethyl acetate fraction of *A. lucidior*, compared to donepezil. Values are expressed as mean ± S.D.

Based on the promising activity of the ethyl acetate fraction, our research group was interested in exploring the anticholinesterase activity of extracted alkaloids from this fraction. The alkaloid-rich fraction (IC_50_ value of 5.26 ± 0.62 µg/mL) revealed promising activity, being more potent than its mother fraction (IC_50_ value of 6.90 ± 0.58 µg/mL), compared to donepezil (IC_50_ = 3.90 ± 0.72 µg/mL) (Table 3 and Figure 5). To our knowledge, no previous studies have reported budmunchiamine alkaloids’ bioactivity in inhibiting the AChE enzyme. Thus, the current study represented the first evidence of the AChE inhibitory activity of budmunchiamine alkaloids. This further assures the potential of *A. lucidior* as an AChE inhibitor, strongly correlated with its richness in budmunchiamine alkaloids.

### 2.5. Molecular Docking Simulation

Initially, all identified alkaloids in the ethanolic extract of *A. lucidior* were inspected via molecular docking to gain more insights into their differential binding poses towards the AChE active site. In this study, the Molecular Operating Environment (MOE) software validity was confirmed by obtaining a low Root Mean Square Deviation (RMSD) value of 1.86 and a similar binding mode for the superimposed redocked MF2 700 inhibitor (green) over its native one (red), Figure S3. Furthermore, it was observed that the co-crystallized inhibitor (MF2 700) stabilized inside the AChE binding site by forming one covalent bond with Ser200 and three H-bonds with Gly118, Gly119 and Ala201 amino acids.

The docking results of *A. lucidior* alkaloids revealed that normethyl budmunchiamine K and budmunchiamine L5 were the most promising candidates, compared to donepezil and docked MF2 700, showing high affinities towards the binding pocket of AChE with binding scores of −10.24 (RMSD = 1.97) and −9.81 kcal/mol (RMSD = 1.82), respectively (Table 4). Surprisingly, the aforementioned binding scores for both alkaloids were recorded without needing to bind either amino acid for stabilization, which indicates their promising and recommended intrinsic activities. However, donepezil was found to form only a pi–pi interaction with the Tyr334 amino acid of the AChE binding pocket at 3.97 Å. Its binding score was −8.03 kcal/mol (RMSD = 1.64). Furthermore, the docked MF2 700 inhibitor binding score was found to be −7.88 kcal/mol (RMSD = 1.29). It formed two H-bonds with Gly118 and Gly119 amino acids at 3.09 and 2.90 Å, respectively. Based on the above data, we can observe the superior binding scores of normethyl budmunchiamine K and budmunchiamine L5, compared to both donepezil and the co-crystallized inhibitor (MF2 700) as two reference standards. This recommends their promising affinities and the corresponding intrinsic activities as well.

### 2.6. Molecular Dynamics (MD) Simulations

**Figure 6:**
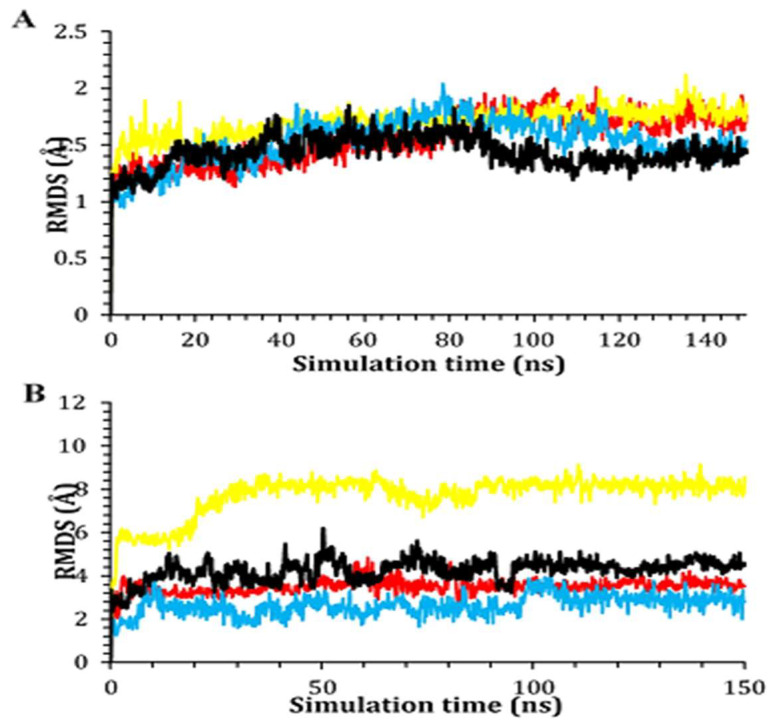
The RMSD of (**A**) the best two protein complexes and (**B**) the best two ligands within the binding pocket of acetylcholinesterase (1OCE) compared to both donepezil and the co-crystallized MF2 700 inhibitor as a function of simulation time (150 ns). Normethyl budmunchiamine K, budmunchiamine L5, donepezil and the co-crystallized MF2 700 are colored red, yellow, blue and black, respectively.

To better understand the differential thermodynamic behavior of the studied AChE complexes at near-physiological conditions, molecular dynamic simulation was further executed. Notably, molecular dynamics is a scientific-based approach to support the reliability of molecular docking obtained binding interactions [52]. First, the RMSD which is essential to describe the deviation degree for each structure compared to its initial position quantitatively was performed. This is important to validate the stability of the examined system during the 150 ns of the simulation time. The RMSD of the four docked AChE complexes showed stable behaviors over the simulation time with very promising values of less than 2 Å. The four complexes showed fluctuations within the range of 1 Å indicating very stable behaviors as well (Figure 6A). On the other hand, the ligands’ RMSD within the AChE receptor was calculated with respect to the 150 ns of the simulation time (Figure 6B). Regarding the individual behavior of each ligand within the AChE (1OCE) receptor pocket, it was recorded that ligand normethyl budmunchiamine K behavior was superior to that of the co-crystallized MF2 700 inhibitor, where it achieved a highly stable behavior (RMSD < 4 Å) inside the receptor pocket till the end of the simulation time. However, ligand budmunchiamine L5 showed a less stable behavior and deviates after 20 ns of the simulation to a higher RMSD value. Moreover, donepezil showed stable behavior regarding the receptor pocket from the start till the end of the simulation. Notably, it showed higher fluctuations with an RMSD < 4 Å. Finally, the co-crystallized MF2 700 ligand showed moderate stability within the receptor pocket. It fluctuated within the range of 4 Å from the start till the end of the simulation time.

Based on the above, it is worth mentioning that normethyl budmunchiamine K and donepezil were the most stable members within the receptor pocket of AChE. They showed closely similar behaviors to that of the co-crystallized MF2 700 inhibitor. However, the fluctuations of normethyl budmunchiamine K were lower than that of donepezil indicating a more stable behavior, greater affinity, and expected intrinsic activity accordingly.

#### 2.6.1. Histograms Analysis

**Figure 7:**
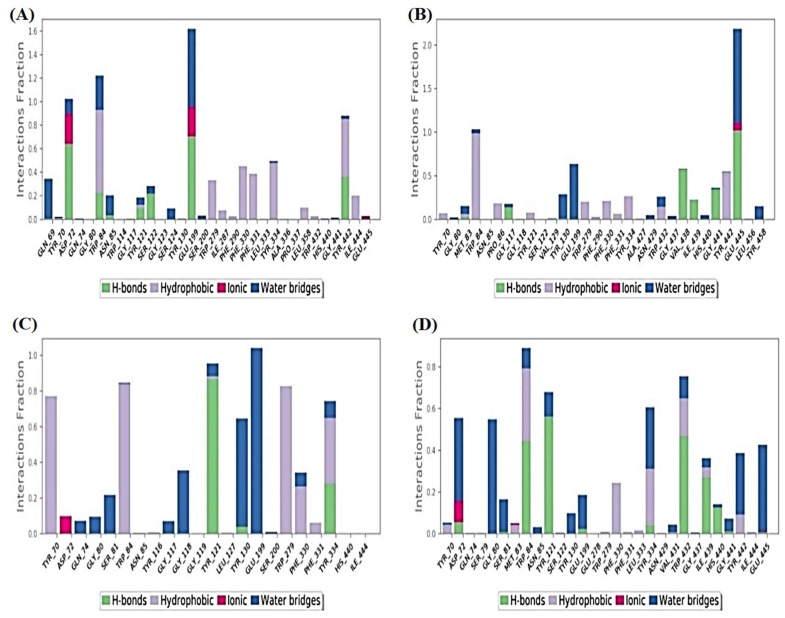
Histograms describing the binding interactions fraction between the protein and its ligand for: (**A**) normethyl budmunchiamine K-1OCE; (**B**) budmunchiamine L5-1OCE; (**C**) donepezil-1OCE and (**D**) MF2 700-1OCE complexes.

The protein–ligand binding interactions fraction of the four studied complexes were described using each histogram as depicted in Figure 7. With respect to the normethyl budmunchiamine K-1OCE complex (Figure 7A), it was noted that Glu199 was the main amino acid that contributed to the binding interactions (160%) as H-bonds, water-bridged hydrogen bonds and ionic bonds (70, 60 and 30%, respectively). Moreover, both Trp84 and Asp72 amino acids contributed 120 and 100% of the binding interactions, respectively. Trp84 interacted with this ligand through hydrophobic bonds (80%), H-bonds (20%), and water-bridged hydrogen bonds (20%), while Asp72 binding interactions to ligand were through H-bonds (60%), ionic bonds (30%) and water bridged hydrogen bonds (10%).

While the histogram of the budmunchiamine L5-1OCE complex (Figure 7B) showed that Glu445 amino acid was the superior one in the binding interactions (220%) divided as water-bridged hydrogen bonds (110%), H-bonds (100%) and ionic bonds (10%). Then, came Trp84 as the second amino acid in the binding interactions (105%) through the formation of hydrophobic (100%) and water-bridged hydrogen bonds (5%).

However, the histogram of the donepezil-1OCE complex (Figure 7C) indicated that Glu199 was the most important amino acid in the interactions with donepezil (105%) through the formation of only water-bridged hydrogen bonds. Tyr121 also contributed with 98% as H-bonds (85%), water-bridged hydrogen bonds (10%) and hydrophobic bonds (3%). It is also worth mentioning that Trp84, Trp279 and Tyr70 amino acids contributed about 80% through hydrophobic interactions.

Furthermore, regarding the MF2 700-1OCE complex (Figure 7D), it was clear that Trp84 amino acid was the main contributing one in the interactions fraction to the co-crystallized MF2 700 inhibitor (90%). These interactions were in the form of H-bonds (45%), hydrophobic (35%) and water-bridged hydrogen bonds (10%). On the other hand, Trp432 contributed 80% to the interactions through H-bonds (50%), hydrophobic (20%), and water-bridged hydrogen bonds (10%). Based on the above, we can conclude that both Glu199 and Trp84 amino acids were the most important ones in the binding interactions of the studied complexes.

#### 2.6.2. Heat Maps Analysis

**Figure 8:**
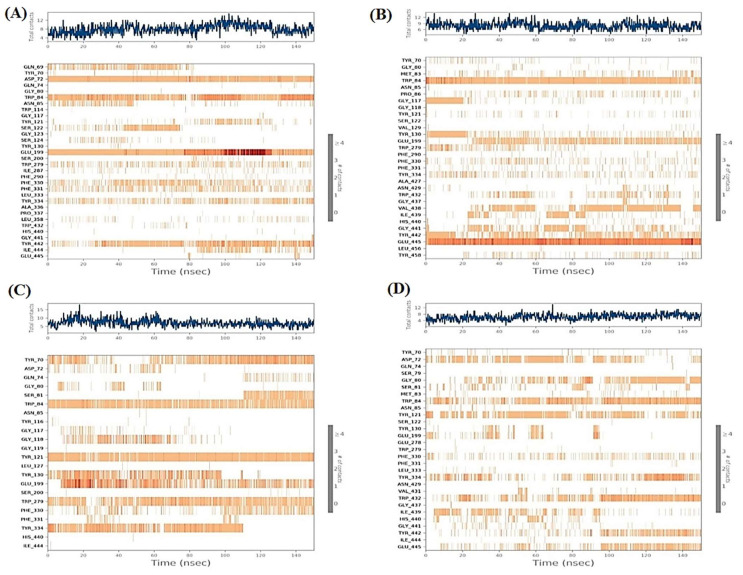
Heat map showing the total number of protein-ligand interactions with respect to the simulation time of 150 ns for: (**A**) normethyl budmunchiamine K-1OCE; (**B**) budmunchiamine L5-1OCE; (**C**) donepezil-1OCE and (**D**) MF2 700-1OCE complexes.

The total number of contacts of the four studied complexes with respect to the time of simulation (150 ns) are described in Figure 8. Analyzing the heat map of normethyl budmunchiamine K within the binding pocket of acetylcholinesterase (1OCE), it was obvious that Glu199 interactions were from the start till the end of the simulation time (100%). While the interactions of Asp72 and Trp84 amino acids were >90 and 80%, respectively, regarding the simulation time (Figure 8A). However, the heat map of budmunchiamine L5 showed that Glu445 amino acid interactions were all over the time of simulation (100%) and the interactions of Trp84 amino acid were >90% of the simulation time (Figure 8B). Moreover, the donepezil heat map (Figure 8C) showed that Glu199 interactions started after 10 ns with >60% contributions regarding the simulation time. Moreover, Trp84, Tyr121, and Trp279 amino acids showed >90, 80 and 70% contributions, respectively, of the simulation time. While Tyr70 amino acid contributed with >60% and showed no contributions from 30–50 ns. Finally, the co-crystallized MF2 700 inhibitor heat map (Figure 8D) clarified that Trp84 contributed to the interactions with about >70% of the simulation time. However, Trp432 amino acid contributions were more obvious after 95 ns with > 50% interactions with respect to the simulation time.

### 2.7. MD Trajectory Analysis and Prime MM-GBSA Calculations

The average MM-GBSA binding energy was calculated using the thermal_mmgbsa.py python script of Schrodinger to measure covalent binding, hydrogen-bonding, coulomb, generalized born electrostatic solvation, lipophilic and Van der Waals energies. The calculated energies for normethyl budmunchiamine K and budmunchiamine L5 besides donepezil and MF2 700 at the active site of the acetylcholinesterase (1OCE) receptor pocket are represented in Table 5.

According to the represented data in Table 5 regarding the calculated energies at the active site of the acetylcholinesterase (1OCE) receptor, we could observe that the ΔG Binding energies of both alkaloids, normethyl budmunchiamine K and budmunchiamine L5 (−90.69 and −76.83 kcal/mol, respectively) are greatly higher than those of the two reference standards, revealing that normethyl budmunchiamine K was the most stable one within the binding pocket of acetylcholinesterase with a much greater value, compared to all other compounds. This was found to be in great agreement with the molecular docking and molecular dynamics simulation results as well. Moreover, normethyl budmunchiamine K achieved superior covalent binding and hydrogen-bonding values (5.14 and −2.26 kcal/mol), compared to all the studied compounds. The Coulomb, Lipophilic, Generalized Born electrostatic solvation and Van der Waals energies of budmunchiamine L5 were higher in values (−150.52, −35.25, 180.17, and −73.12 kcal/mol, respectively), compared to the other tested compounds.

## 3. Materials and Methods

### 3.1. Plant Material, Extraction and Fractionation of Plants

The leaves of *Albizia lucidior* (Steud.) I.C.Nielsen and *Albizia procera* (Roxb.) Benth. (Family Fabaceae) were collected, in the flowering stage, in March 2019 from Zoological garden and Mazhar Botanical Garden, Giza, Egypt, respectively, and identified by Agr. Eng. Therese Labib, consultant of plant taxonomy at the Ministry of Agriculture and ex. Director of El-Orman Botanical Garden, Giza. A voucher specimen (No. 4.7.2019) was kept at the Herbarium of Pharmacognosy Department, Faculty of Pharmacy, Cairo University.

The air-dried powdered leaves of the studied species (1500 g, each) were extracted with 90% ethanol (8 × 1 L). The extracts of *A. lucidior* and *A. procera* were evaporated under reduced pressure in a rotary evaporator (Büchi, Switzerland), yielding 16.67% and 19.33%, respectively (expressed as the weight of the extract relative to the weight of the initial plant material). An aliquot (150 g) of *A. lucidior* ethanolic extract was suspended in distilled water followed by fractionation with petroleum ether, dichloromethane, ethyl acetate, and *n*-butanol saturated with water. Each fraction was dried separately and weighed, yielding 25, 19, 33 and 42 g, respectively.

### 3.2. In Vitro Acetylcholinesterase Assay

Acetylcholinesterase inhibitory activity of the tested samples was evaluated by Ellman’s microplate assay with slight modifications [53]. Absorbances were measured using a microplate reader (BioTek Instruments, Inc., Winooski, VT, USA) at 412 nm after 30 min of initiation of enzymatic reaction. Each test was conducted in triplicate. Donepezil was used as a positive control. The results were expressed as the percentage inhibition (%) and IC_50_ values (µg/mL) of each sample were also calculated.

### 3.3. Determination of Total Phenolic and Flavonoid Contents

Total phenolic (TPC) and total flavonoid (TFC) contents of tested samples were determined spectrophotometrically using Folin–Ciocalteu and Aluminum chloride assays, respectively. The absorbance of the color produced was measured at 630 nm for TPC [54] and 415 nm for TFC [55] on a microplate reader (FluoStar Omega, bmg labtech, Ortenberg, Germany).

### 3.4. UHPLC-QTOF-MS/MS Profiling of the Crude Extracts and Fractions

Ultra-high-performance liquid chromatograms (UHPLC) were obtained on an Agilent LC–MS system composed of an Agilent 1290 Infinity II UHPLC coupled to an Agilent 6545 ESI-Q-TOF-MS in both negative and positive modes, aliquots (1 µL) of ethanolic extracts (2 mg/mL in MeOH) and fractions (0.5 mg/mL in MeOH) were analyzed on a Kinetex phenyl-hexyl (1.7 µm, 2.1 × 50 mm) column eluted with 1 min isocratic elution of 90% A (A: 100% H_2_O + 0.1% formic acid) followed by 6 min linear gradient elution to 100% B (95% MeCN + 5% H_2_O + 0.1% formic acid) with a flow rate of 0.4 mL/min. ESI conditions were set with the capillary temperature at 320 °C, source voltage at 3.5 kV and a sheath gas flow rate of 11 L/min. Ions detected in the full scan at an intensity above 1000 counts at 6 scans/s, with an isolation width of 1.3 ~*m*/*z*, a maximum of 9 selected precursors per cycle and using ramped collision energy (5× *m*/*z*/100 + 10 eV). Purine C_5_H_4_N_4_ [M + H]^+^ ion (*m*/*z* 121.050873) and hexakis (1H,1H,3H-tetrafluoropropoxy)-phosphazene C_18_H_18_F_24_N_3_O_6_P_3_ [M + H]^+^ ion (*m*/*z* 922.009798) were used as internal lock masses for positive mode while TFA C_2_HF_3_O_2_[M − H]^−^ ion (*m*/*z* 112.985587) and hexakis (1H,1H,3H-tetrafluoropropoxy)-phosphazene C_18_H_18_F_24_N_3_O_6_P_3_ [M + TFA − H]^−^ ion (*m*/*z* 1033.988109) were used as internal lock masses for negative mode.

The .mzXML files were imported and processed with MZmine 2 v2.53 [56] with the following workflow: (i) Mass Detection: MS^1^ noise level, 1E3; MS^2^ noise level, 1E2. (ii) ADAP chromatogram builder: MS-level, 1; min group size in no. of scans, 2; group intensity threshold, 2E4; min highest intensity, 5E3; *m*/*z* tolerance, 0.01 *m*/*z*. (iii) Chromatogram deconvolution: Local minimum search algorithm (iv) Isotopic peaks grouper: *m*/*z* tolerance, 0.01 *m*/*z*; RT tolerance, 0.05 min; monotonic shape, yes; maximum charge, 2; representative isotope, lowest *m*/*z*. (v) peak alignment: *m*/*z* tolerance, 0.02 *m*/*z*; weight for *m*/*z*, 75; RT tolerance, 0.2 min; weight for RT, 25. (vi) Peak list rows filter: only features with accompanying MS^2^ data and their retention time between 0 and 9.0 min were kept. (vii) Duplicate peak filter: filter mode, old average; *m*/*z* tolerance, 0.02 *m*/*z*; RT tolerance, 0.5 min. The resulting feature lists were exported to the GNPS-compatible format, using the dedicated “Export for GNPS” built-in options.

### 3.5. GNPS Feature-Based Molecular MS/MS Network

Using the Feature-Based Molecular Networking (FBMN) workflow (version release_28.2) [57] on GNPS, a molecular network was created. The resulting aligned list of features was exported in an mgf file besides their feature quantification table in csv format. The values of feature quantification table were uploaded onto the FBMN page of GNPS. MS^2^ spectra were filtered, all MS/MS fragment ions within ±17 Da of the precursor *m*/*z* were removed, and only the top 5 fragment ions in the ±50 Da window through the spectrum were utilized. The precursor and fragment ion masses were both set to 0.02 Da. Edges of the molecular network were filtered to have a cosine score above 0.7 and more than 5 matched peaks between the connected nodes. The edges between two nodes were kept in the network and only if each of the nodes appeared in each other’s respective top 10 most similar nodes. The size of clusters in the network was set to a maximum of 100. The molecular networks were visualized using Cytoscape 3.9.1. [58].

### 3.6. Preparation and Purification of Alkaloid-Rich Fraction

The alkaloids were extracted from *A. lucidior* ethyl acetate fraction according to [59]. An aliquot of the alkaloid extract (850 mg) was chromatographed on a silica gel 60 (40 g, 63–200 μm, Merck, Darmstadt, Germany) in a column (3 cm D × 15 cm L), using gradient elution with petroleum ether: dichloromethane: diethylamine mixtures (7:2:1, 6:3:1, 5:4:1, 3:6:1 *v*/*v*/*v*). Fractions (5 mL each) were collected and monitored by TLC using a solvent system (petroleum ether: dichloromethane: diethylamine, 5:4:1 *v*/*v*/*v*). Fractions with similar chromatographic patterns were pooled together, concentrated under reduced pressure and weighed. Based on chromatographic monitoring, five fractions (1–5) were obtained. Fractions 3–4 (117 mg), eluted with petroleum ether: dichloromethane: diethylamine 7:2:1 till 3:6:1 *v*/*v*/*v*, presented the alkaloid-rich fraction and was subjected to biological evaluation. Other fractions exhibited less quality with the occurrence of other non-alkaloidal compounds, and so were discarded in the current study.

### 3.7. Docking Studies and Validation of the MOE Software

A general molecular docking study was carried out for all identified alkaloids from *A. lucidior* in positive mode using the MOE 2019.012 suite [60]. Both donepezil and the co-crystallized inhibitor (MF2 700) were inserted as two reference standards as well. In order to validate the MOE program software and consider the obtained docking results, the co-crystallized inhibitor (MF2 700) of the acetylcholinesterase receptor was redocked inside its binding pocket. Then, the chemical structures of the identified alkaloids from *A. lucidior*, besides donepezil were downloaded as smiles from the PubChem database. Each one was introduced individually to the MOE window to be prepared for docking as described earlier [61]. Furthermore, all of the prepared alkaloids were inserted into one database together with the co-crystallized MF2 700 inhibitor and saved for the docking step as an MDB file. The X-ray structure of the acetylcholinesterase protein (PDB ID:1OCE) was extracted from the Protein Data Bank (PDB) website. It was opened using the MOE window, studied carefully using the sequence editor, and the co-crystallized ligand-protein interactions were investigated as well. Finally, it was prepared for the docking process by applying the previously discussed steps [62]. The previously built database was inserted in place of the ligand during the process of general docking using the ligand site as the docking site. All steps of the docking process methodology were followed as described before in detail [63,64]. The best-docked complexes based on their score values, RMSD, and binding modes were selected for further investigations.

### 3.8. Molecular Dynamics Simulations

The MD simulations were carried out using the Desmond simulation package of Schrödinger LLC [65]. The NPT ensemble with a temperature of 300 K and a pressure of 1 bar was applied in all runs. The simulation length was 150 ns with a relaxation time 1 ps for the ligands. The OPLS3 force field parameters were used in all simulations [66]. The cutoff radius in Coulomb interactions was 9.0 Å. The orthorhombic periodic box boundaries were set 10 Å away from the protein atoms. The water molecules were explicitly described using the transferable intermolecular potential with three points (TIP3P) model [67,68]. The salt concentration was set to 0.15 M NaCl and was built using the System Builder utility of Desmond [69]. The Martyna–Tuckerman–Klein chain coupling scheme with a coupling constant of 2.0 ps was used for the pressure control and the Nosé–Hoover chain coupling scheme for the temperature control [70,71]. Nonbonded forces were calculated using a RESPA integrator where the short-range forces were updated every step and the long-range forces were updated every three steps. The trajectories were saved at 20 ns intervals for analysis. The behavior and interactions between the ligands and protein were analyzed using the Simulation Interaction Diagram tool implemented in the Desmond MD package. The stability of MD simulations was monitored by looking at the RMSD of the ligand and protein atom positions in time.

### 3.9. MD Trajectory Analysis and Prime MM-GBSA Calculations

The simulation interactions diagram panel of Maestro software [65] was used to monitor interactions contribution in the ligand–protein stability. The molecular mechanics generalized born/solvent accessibility (MM–GBSA) was performed to calculate the ligand binding free energies and ligand strain energies for docked metabolites over the last 25 ns with thermal_mmgbsa.py python script provided by Schrodinger which takes a Desmond trajectory file, splits it into individual snapshots, runs the MM-GBSA calculations on each frame, and outputs the average computed binding energy.

### 3.10. Statistical Analysis

The data were presented as mean ± standard deviation (S.D.). One-way analysis of variance (ANOVA) was used, followed by Tukey’s post hoc test (*p* < 0.05) to show the differences between the groups. Denoting the statistically significant levels with different letters is a way to summarize the differences between the extracts. If the two extracts share at least one letter, it means that they are not significantly different.

## 4. Conclusions

Based on the demand for new acetylcholinesterase inhibitors for AD treatment, this work aimed to explore the anticholinesterase potential of *A. lucidior* and *A. procera* leaves. *A. lucidior* ethanolic extract was revealed to be a new promising candidate, with its enzyme inhibitory efficacy mainly localized in its ethyl acetate fraction. We then applied UHPLC-ESI-QTOF-MS/MS-Based Molecular Networking to gain more insights into the differential chemical composition between the two studied species and correlate it to their anticholinesterase activity. MS/MS-Based Molecular Networking characterized the abundance of budmunchiamine alkaloids in *A. lucidior* extract and its ethyl acetate fraction, which were lacking in *A. procera* extract. The molecular networking also unearthed four new putative alkaloids in *A. lucidior*. Further, in vitro inspection of the alkaloid-rich fraction of *A. lucidior* revealed the most potent activity versus the total ethanolic extract and ethyl acetate fraction, in agreement with molecular networking results. Thus, budmunchiamine alkaloids were highlighted for the first time as possible candidate metabolites beyond *A. lucidior* bioactivity, which will attract more focus towards this class of compounds. In this context, normethyl budmunchiamine K revealed higher binding affinity and stability within the binding pocket of acetyl cholinesterase, compared to donepezil; a standard drug for AD treatment, based on molecular-docking and molecular dynamics simulations as well as molecular mechanics generalized born/solvent accessibility (MM–GBSA) calculations.

Conclusively, this study represents the first for the anti-cholinesterase activities and chemical profiling of *A. lucidior* and *A. procera* leaves, alongside the potential of normethyl budmunchiamine K as a lead drug for designing new AChE inhibitors for Alzheimer’s disease treatment. Yet, further in vivo and clinical studies should now follow to confirm the treatment effectiveness of this metabolite in Alzheimer’s disease.

## Figures and Tables

**Table 1:** Acetylcholinesterase inhibitory activity, represented by IC_50_ of plant extracts and fractions.

Extract/Fraction	IC_50_ (µg/mL)
Ethanolic extract of *A. procera*	43.50 ± 2.10 ^e^
Ethanolic extract of *A. lucidior*	24.89 ± 1.60 ^d^
Petroleum ether fraction of *A. lucidior*	ND
Dichloromethane fraction of *A. lucidior*	17.40 ± 1.30 ^c^
Ethyl acetate fraction of *A. lucidior*	6.90 ± 0.96 ^b^
*n*-butanol fraction of *A. lucidior*	>125 ^f^
Donepezil	3.90 ± 0.72 ^a^

ND, not detected. Different superscript letters (a–f) mean statistically significant differences in the same column (*p* < 0.05) by Tukey’s test. Donepezil is a positive control.

**Table 3:** Acetylcholinesterase inhibitory activity of *A. lucidior* ethyl acetate fraction *vs* its alkaloid-rich fraction.

Tested Samples	IC_50_ (µg/mL)
Ethyl acetate fraction	6.90 ± 0.96 ^b^
Alkaloid-rich fraction	5.26 ± 0.62 ^a^
Donepezil	3.90 ± 0.72 ^a^

Different superscript letters mean statistically significant differences in the same column (*p* < 0.05) by Tukey’s test. Donepezil is a positive control.

**Table 4:** Binding scores, RMSD, 3D binding interactions, and 3D positioning of the most promising alkaloids of *A. lucidior* inside the binding pocket of acetylcholinesterase, compared to donepezil and MF2 700 inhibitor.

Compounds	^a^ S RMSD	2D Interaction	3D Interaction	3D Positioning
Normethyl budmunchiamine K	−10.24 1.97	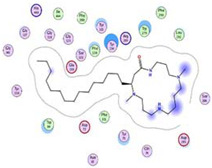	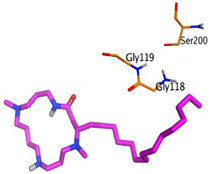	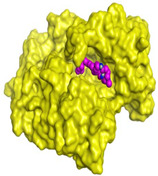
Budmunchiamine L5	−9.81 1.82	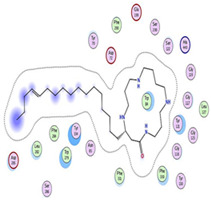	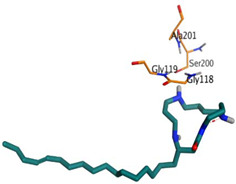	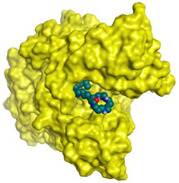
Donepezil	−8.03 1.64	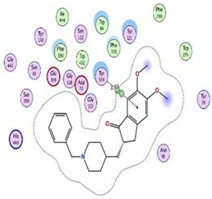	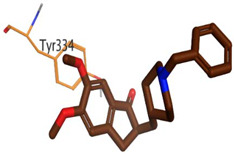	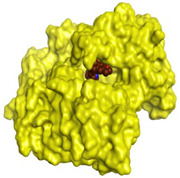
MF2 700	−7.88 1.29	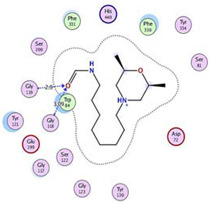	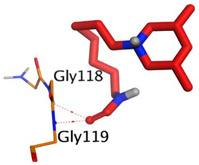	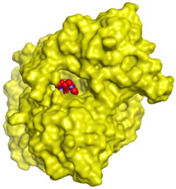

^a^ S: Score of a compound within the protein binding pocket (Kcal/mol).

**Table 5:** Prime MM-GBSA energies for the best two alkaloids compared to both donepezil and the co-crystallized MF2 700 inhibitor binding at the active site of the 1OCE receptor pocket.

Complexes	Normethylbudmunchiamine K-1OCE	Budmunchiamine L5-1OCE	Donepezil -1OCE	MF2 700 -1OCE
ΔG Binding	−90.69	−76.83	−68.99	−64.55
Coulomb	−32.56	−150.52	−44.38	−51.57
Covalent	5.14	3.72	1.36	1.37
H-bond	−2.26	−1.81	−0.63	−1.28
Lipo	−32.61	−35.25	−30.90	−23.44
Bind Packing	−6.94	0	−7.66	0
Solv_GB	48.55	180.17	65.41	54.60
VdW	−70.01	−73.12	−52.18	−44.22
St. Dev.	7.02	4.88	4.51	5.62

Coulomb: Coulomb energy; Covalent: Covalent binding energy; H-bond: Hydrogen-bonding energy; Lipo: Lipophilic energy; Solv_GB: Generalized Born electrostatic solvation energy; VdW: Van der Waals energy; and St. Dev.: standard deviation.

## Data Availability

Not applicable.

## Supplementary Materials

The following supporting information can be downloaded at: https://www.mdpi.com/article/10.3390/plants11233286/s1, Table S1. Identified metabolites in the negative ion mode of ethanolic extracts of A. lucidior and A. procera leaves using UHPLC-MS/MS. Figure S1. GNPS molecular network of two Albizia spp. ethanolic extracts in the positive ion mode. Figure S2. GNPS molecular network of two Albizia spp. ethanolic extracts in the negative ion mode. Figure S3. Superimposition of the redocked MF2 700 inhibitor (green) over its native one (red).
