# Supplementary material for: Anticholinesterase Activity of Budmunchiamine Alkaloids Revealed by Comparative Chemical Profiling of Two Albizia spp., Molecular Docking and Dynamic Studies
Source: Plants (Basel). 2022 Nov 29;11(23):3286. doi: 10.3390/plants11233286 (PMC9738009; doi:10.3390/plants11233286)
Supplement: Supplementary file 1 [file plants-11-03286-s001.zip › plants-1964872-supplementary.pdf]

Supporting Information

Anticholinesterase Activity of Budmunchiamine Alkaloids Revealed by Comparative Chemical Profiling of Two *Albizia* spp., Molecular Docking and Dynamic Studies

Mai E. Hussein [1,*,†], Osama G. Mohamed [1,2,†], Ahlam M. El-Fishawy [1], Hesham I. El-Askary [1], Ahmed A. Hamed [3], Marwa M. Abdel-Aziz [4], Radwan Alnajjar [5,6,7], Amany Belal [8], Ahmed M. Naglah [9,10], Abdulrahman A. Almehizia [9], Ahmed A. Al-Karmalawy [11], Ashootosh Tripathi [2,12] and Amira S. El Senousy [1]

1 Pharmacognosy Department, Faculty of Pharmacy, Cairo University, Kasr el Aini St., Cairo 11562, Egypt
2 Natural Products Discovery Core, Life Sciences Institute, University of Michigan, Ann Arbor, MI 48109, USA
3 Microbial Chemistry Department, National Research Centre, 33 El-Buhouth Street, Dokki, Giza 12622, Egypt
4 Regional Center for Mycology and Biotechnology (RCMB), Al-Azhar University, Cairo 11651, Egypt
5 Department of Chemistry, Faculty of Science, University of Benghazi, Benghazi, Libya
6 PharmD, Faculty of Pharmacy, Libyan International Medical University, Benghazi, Libya
7 Department of Chemistry, University of Cape Town, Rondebosch 7701, South Africa
8 Medicinal Chemistry Department, Faculty of Pharmacy, Beni-Suef University, Beni-Suef 62514, Egypt
9 Drug Exploration and Development Chair (DEDC), Department of Pharmaceutical Chemistry, College of Pharmacy, King Saud University, Riyadh 11451, Saudi Arabia
10 Peptide Chemistry Department, National Research Centre, Dokki, Cairo 12622, Egypt
11 Pharmaceutical Chemistry Department, Faculty of Pharmacy, Ahram Canadian University, 6th of October City, Giza 12566, Egypt
12 Department of Medicinal Chemistry, College of Pharmacy, University of Michigan, Ann Arbor, MI 48109, USA
* Correspondence: mai.husein@pharma.cu.edu.eg
† These authors contributed equally to this work.

List of Supporting Information

Table S1: Identified metabolites in the negative ion mode of ethanolic extracts of *A. lucidior* and *A. procera* leaves using UHPLC-MS/MS 3-7

List of Figures

Figure S1. GNPS molecular network of two *Albizia* spp. ethanolic extracts in the positive ion mode........ 8
Figure S2. GNPS molecular network of two *Albizia* spp. ethanolic extracts in the negative ion mode........ 9
Figure S3. Superimposition of the redocked MF2 700 inhibitor (green) over its native one (red)........ 10

Table S1: Identified metabolites in the negative ion mode of ethanolic extracts of *A. lucidior* and *A. procera* leaves using UHPLC-MS/MS

No.	R_t (min)	Name	Ion m/z ppm	Molecular Formula	MS/MS fragmentation product ions	Ref	Al	Ap
1	0.41	Dicaffeoylhexaric acid	533.1718	$C_{23}H_{33}O_{14}^-$	209.0653; 191.0561 85.0300	[1]	√	-
2	0.42	Quinic acid	191.0563	$C_7H_{11}O_6^-$	173.0447; 127.0396 93.0344; 85.0293	[2]	√	√
3	0.43	Xylonic acid	165.0403	$C_5H_9O_6^-$	147.0275; 135.0269 105.0185; 87.0084 75.0086	[2]	√	√
4	0.49	Gallic acid glucoside	331.0686	$C_{13}H_{15}O_{10}^-$	211.0237; 169.0147 151.0026; 125.0241	[1]	-	√
5	0.55	Gallic acid	169.0143	$C_7H_5O_5^-$	125.0258; 107.0153 97.0309; 79.0207	[3]	-	√
6	0.76	Protocatechuic acid	153.0190	$C_7H_5O_4^-$	109.0293	[4]	√	√
7	0.77	Vanillic acid-*O*-glucoside	329.0885	$C_{14}H_{17}O_9^-$	209.0452; 167.0345 125.0246; 123.0449	[1]	-	√
8	0.80	Catechol	109.0287	$C_6H_5O_2^-$	108.0217; 91.0191 81.0335; 66.0361	[5]	√	-
9	0.88	Gentisic acid-5-*O*-xyloside	285.0619	$C_{12}H_{13}O_8^-$	152.0114; 108.0216	[6]	√	-
10	0.91	Digalloyl-glucose	483.0779	$C_{20}H_{19}O_{14}^-$	313.0553; 271.0455 169.0137	[7]	-	√
11	0.99	Caffeic acid glucuronide	355.0685	$C_{15}H_{15}O_{10}^-$	209.0272;191.0545 161.0240; 85.0292	[2]	√	-
12	1.05	Methyl gallate	183.0301	$C_8H_7O_5^-$	168.0046; 124.0164	[3]	-	√
13	1.06	*p*-coumaric acid-4-*O*-glucoside	325.0936	$C_{15}H_{17}O_8^-$	163.0395; 119.0499	[8]	√	√
14	1.07	Aesculin	339.0731	$C_{15}H_{15}O_9^-$	177.0195	[8]	√	-
15	1.22	Ferulic acid glucoside	355.1033	$C_{16}H_{19}O_9^-$	309.0453; 193.0499 178.0266; 134.0374	[9]	-	√
16	1.34	Shikimic acid	173.0452	$C_7H_9O_5^-$	136.9332; 111.0452 93.0360; 85.0303 83.0133	[10]	√	√
17	1.36	Caffeoylquinic acid	353.0880	$C_{16}H_{17}O_9^-$	191.0556; 173.0445 135.0446; 85.0297	[8]	√	√
18	1.36	Umbelliferone	161.0241	$C_9H_5O_3^-$	133.0288	[11]	√	-
19	1.37	Caffeic acid	179.0352	$C_9H_7O_4^-$	135.0459; 89.0028	[1]	√	√
20	1.46	Esculetin	177.0193	$C_9H_5O_4^-$	149.0253; 133.0296 105.0347; 89.0399	[3]	√	√

Table S2: Identified metabolites in the negative ion mode of ethanolic extracts of *A. lucidior* and *A. procera* leaves using UHPLC-MS/MS

No.	R_t (min)	Name	Ion m/z ppm	Molecular Formula	MS/MS fragmentation product ions	Ref	Al	Ap
21	1.92	Rosmarinic acid-*O*-glucoside	521.2047	$C_{24}H_{25}O_{13}^{-}$	359.1511; 344.1271	[12]	-	√
22	2.03	Syringic acid	197.0457	$C_9H_9O_5^{-}$	169.0142; 124.0168	[7]	-	√
23	2.09	Feruloyl quinic acid	367.1042	$C_{17}H_{19}O_9^{-}$	193.0521;173.0454	[13]	-	√
24	2.10	*p*-coumaroyl quinic acid	337.0934	$C_{16}H_{17}O_8^{-}$	191.0556; 163.0394<br>119.0491; 93.0342	[12]	√	√
25	2.35	*p*-coumaric acid	163.0401	$C_9H_7O_3^{-}$	119.0510; 93.0350<br>91.0568	[1]	√	√
26	2.45	Myricetin-3-*O*-rutinoside	625.1415	$C_{27}H_{29}O_{17}^{-}$	317.0293; 316.0234<br>287.0917; 271.0252<br>178.9991; 151.0043	[14]	-	√
27	2.45	Coumaroyl-*O*-galloyl glucose	477.1040	$C_{22}H_{21}O_{12}^{-}$	313.0558; 169.0151	[7]	-	√
28	2.48	Hexosylrutin	771.1992	$C_{33}H_{39}O_{21}^{-}$	609.1453; 463.0886<br>301.0346; 300.0273	[9]	-	√
29	2.49	Methyl caffeoyl quinate	367.1033	$C_{17}H_{19}O_9^{-}$	191.0564; 179.0349<br>161.0249; 135.0452	[13]	√	√
30	2.51	Quercetin rhamnosyl-rutinoside	755.2053	$C_{33}H_{39}O_{20}^{-}$	609.1423; 301.0342<br>300.0280	[9]	√	-
31	2.51	Myricetin-3-*O*-glucoside	479.0837	$C_{21}H_{19}O_{13}^{-}$	317.0287; 316.0281<br>271.0235; 178.9972<br>151.0023	[1]	-	√
32	2.51	Myricetin-3-*O*-glucuronide	493.0630	$C_{21}H_{17}O_{14}^{-}$	317.0297; 299.0184<br>271.0262; 178.9974<br>151.0025	[15]	-	√
33	2.54	Naringenin-7-*O*-glucoside	433.1151	$C_{21}H_{21}O_{10}^{-}$	313.0707; 271.0618<br>193.0381; 119.0493	[10]	-	√
34	2.62	Myricetin-3-*O*-arabinoside	449.0760	$C_{20}H_{17}O_{12}^{-}$	317.0326; 316.0229<br>287.0182; 271.0238<br>178.9977	[14]	-	√
35	2.62	Kaempferol-rhamnosyl-galactoside-rhamnoside	739.2108	$C_{33}H_{39}O_{19}^{-}$	593.1507; 285.0403<br>284.0334; 255.0299	[9]	√	√
36	2.65	Quercetin-3-*O*-sambubioside	595.1318	$C_{26}H_{27}O_{16}^{-}$	301.0348; 300.0287<br>271.0252; 255.0304<br>178.9990; 151.0041	[16]	√	√
37	2.69	Kaempferol-3-(2G-xylosylrutinoside)	725.1944	$C_{32}H_{37}O_{19}^{-}$	575.1360; 285.0391<br>284.0327; 255.0304<br>227.0334; 151.0034	[9]	√	√
38	2.69	Rutin	609.1468	$C_{27}H_{29}O_{16}^{-}$	445.0696; 343.0456<br>301.0351; 300.0275<br>271.0242; 255.0297<br>178.9975; 151.0029	[2]	√	√

Table S3: Identified metabolites in the negative ion mode of ethanolic extracts of *A. lucidior* and *A. procera* leaves using UHPLC-MS/MS

No.	R_t (min)	Name	Ion m/z ppm	Molecular Formula	MS/MS fragmentation product ions	Ref	Al	Ap
39	2.71	Quercetin-*O*-galloyl-glucoside	615.0990	$C_{28}H_{23}O_{16}^-$	463.0890; 313.0557 301.0354; 300.0281 271.0246; 255.0286 169.0143	[2]	√	-
40	2.76	Myricetin-3-*O*-rhamnoside (myricitrin)	463.0894	$C_{21}H_{19}O_{12}^-$	317.0298; 316.0232 287.0202; 271.0248 178.9984	[3]	√	√
41	2.79	Quercetin-3-*O*-glucuronide	477.0684	$C_{21}H_{17}O_{13}^-$	301.0354; 255.0295 178.9984; 151.0033	[15]	-	√
42	2.80	Kaempferol-3-*O*-rutinoside	593.1522	$C_{27}H_{29}O_{15}^-$	285.0403; 284.0323 255.0299; 227.0345 151.0033	[2]	√	√
43	2.85	Quercetin pentose deoxy-hexose	579.1375	$C_{26}H_{27}O_{15}^-$	447.0832; 301.0326 300.0288; 255.0314	[9]	√	-
44	2.85	*p*-Hydroxybenzoic acid	137.0245	$C_7H_5O_3^-$	93.0354; 65.0397	[13]	√	-
45	2.85	Azelaic acid	187.0975	$C_9H_{15}O_4^-$	169.0863; 125.0980 123.0825; 97.0669	[17]	√	-
46	2.93	Kaempferol-3-*O*-sambubioside	579.2086	$C_{26}H_{27}O_{15}^-$	417.1552; 285.0408	[18]	√	-
47	2.94	Quercetin-3-*O*-arabinoside (Avicularin)	433.0789	$C_{20}H_{17}O_{11}^-$	301.0344; 300.0275 271.0241; 255.0301 151.0043	[9]	√	√
48	2.96	Myrciacitrin	477.1030	$C_{23}H_{25}O_{11}^-$	317.0247; 316.0231 287.0188; 178.9986 151.0023	[9]	-	√
49	2.98	Kaempferol-3-*O*-glucuronide	461.0736	$C_{21}H_{17}O_{12}^-$	285.0406; 257.0453 229.0506	[14]	-	√
50	3.00	Quercetin 3-*O*-rhamnoside (Quercitrin)	447.0950	$C_{21}H_{19}O_{11}^-$	301.0356; 300.0281 271.0249; 255.0301 178.9987; 151.0043	[3]	√	√
51	3.06	Ferulic acid	193.0508	$C_{10}H_9O_4^-$	161.0247; 134.0374 133.0298	[1]	√	√
52	3.10	Phloridzin	435.1304	$C_{21}H_{23}O_{10}^-$	273.0775; 167.0353	[3]	-	√
53	3.18	Quercetin benzoyl-glucoside	583.4803	$C_{28}H_{23}O_{14}^-$	463.0891; 301.0322 300.0269; 271.0229 255.0282; 152.0106	[7]	√	-

Table S4: Identified metabolites in the negative ion mode of ethanolic extracts of *A. lucidior* and *A. procera* leaves using UHPLC-MS/MS

No.	Rt (min)	Name	Ion m/z ppm	Molecular Formula	MS/MS fragmentation product ions	Ref	Al	Ap
54	3.18	Kaempferol-3-*O*-rhamnoside	431.0988	$C_{21}H_{19}O_{10}^-$	285.0416; 284.0340 255.0310; 227.0360 151.0036	[2]	√	√
55	3.19	Myricetin	317.0309	$C_{15}H_9O_8^-$	178.9998; 151.0448 137.0250	[9]	-	√
56	3.21	Quercetin-3-*O*-glucosyl-6″-acetate	505.1002	$C_{23}H_{21}O_{13}^-$	329.0664; 301.0343 300.0280; 271.0241 255.0290; 151.0023	[10]	-	√
57	3.21	Myricetin-*O*-(*O*-galloyl)-3-rhamnoside	615.1002	$C_{28}H_{23}O_{16}^-$	463.0895; 317.0315 178.9998;169.0148 151.0047	[3]	-	√
58	3.21	Quercetin-di-*O*-glucoside	625.1202	$C_{27}H_{29}O_{17}^-$	463.0891; 301.0359 300.0284; 255.0315	[1]	√	-
59	3.21	Chrysoeriol-*O*-glucoside	461.1084	$C_{22}H_{21}O_{11}^-$	299.0200	[19]	-	√
60	3.41	Quercetin galloyl rhamnoside	599.1051	$C_{28}H_{23}O_{15}^-$	447.0920; 383.1042 301.0368; 273.0402 178.9991; 151.0041	[2]	-	√
61	3.41	Kaempferol acetyl glycoside	489.1044	$C_{23}H_{21}O_{12}^-$	285.0409; 284.0335 255.0306; 227.0359	[1]	-	√
62	3.43	Quercetin coumaroyl glucoside	609.1263	$C_{30}H_{25}O_{14}^-$	463.0872; 301.0352 300.0276; 271.0238 178.9977; 151.0031	[1]	√	-
63	3.46	Quercetin feruloyl glucoside	639.1359	$C_{31}H_{27}O_{15}^-$	463.0881; 301.0350 300.0276; 271.0248 255.0291; 151.0025	[7]	√	-
64	3.48	Ethyl caffeate	207.0665	$C_{11}H_{11}O_4^-$	179.0352; 161.0250 135.0453; 134.0376	[13]	√	√
65	3.52	*p*-methoxycinnamic acid	177.0563	$C_{10}H_9O_3^-$	162.0317; 145.0296 133.0061;118.0427	[20]	-	√
66	3.55	Quercetin	301.0345	$C_{15}H_9O_7^-$	178.9993; 151.0045 121.0296;107.0147	[1]	√	√
67	3.55	Kaempferol	285.0396	$C_{15}H_9O_6^-$	243.0301; 217.0505 199.0403; 175.0397	[1]	√	√
68	3.60	Trihydroxyoctadienoic acid	327.2182	$C_{18}H_{31}O_5^-$	291.1969; 229.1449 211.1349; 171.1033	[2]	√	√
69	3.77	Trihydroxyoctadecenoic acid	329.2336	$C_{18}H_{33}O_5^-$	229.1452; 211.1348 171.1031	[2]	√	√
70	3.85	Naringenin	271.0623	$C_{15}H_{11}O_5^-$	151.0039; 119.0509 65.0045	[1]	-	√
71	3.85	Dihydroxy palmitic acid	287.2233	$C_{16}H_{31}O_4^-$	269.2144; 241.2189 223.2067	[2]	√	√

Table S5: Identified metabolites in the negative ion mode of ethanolic extracts of *A. lucidior* and *A. procera* leaves using UHPLC-MS/MS

No.	R_t (min)	Name	Ion m/z ppm	Molecular Formula	MS/MS fragmentation product ions	Ref	Al	Ap
72	4.42	Dihydroxyoctadecadienoic acid	311.2223	$C_{18}H_{31}O_4^-$	311.2208; 293.2096 275.2005; 223.1705	[19]	√	√
73	4.51	Epoxy-hydroxyoctadeca-dienoic acid	309.2073	$C_{18}H_{29}O_4^-$	291.1959; 273.1980 221.1541	[19]	√	√
74	4.56	Pinocembrin	255.0665	$C_{15}H_{11}O_4^-$	213.0556; 171.0443 151.0031; 107.0134 83.0136; 65.0037	[1]	-	√
75	4.58	Dihydroxyoctadecenoic acid	313.2386	$C_{18}H_{33}O_4^-$	295.2300; 277.2174 183.1392	[19]	-	√
76	4.98	Sphingolipid conjugate II	564.3315	$C_{27}H_{51}O_9NP^-$	279.2329	[19]	√	-
77	5.00	13-keto-octadeca-9,11-dienoic acid	293.2126	$C_{18}H_{29}O_3^-$	275.2027; 223.1346 195.1396; 179.1463	[5]	√	√
78	5.13	Hydroxyoctadecenoic acid	297.1535	$C_{18}H_{33}O_3^-$	279.9880; 183.0120	[19]	√	√
79	5.17	9-hydroxy-10,12-octadecadienoic acid	295.2276	$C_{18}H_{31}O_3^-$	277.2178; 195.1393 171.1029	[5]	√	√
80	5.20	Sphingolipid conjugate III	566.3478	$C_{27}H_{53}O_9NP^-$	281.2497	[19]	√	-
81	5.76	Hydroxy palmitic acid	271.2275	$C_{16}H_{31}O_3^-$	253.2167; 225.2220	[19]	√	√
82	5.93	Linolenic acid	277.2171	$C_{18}H_{29}O_2^-$	259.2068; 234.1868	[5]	√	√
83	5.96	Yohimbic acid	339.1999	$C_{20}H_{23}N_2O_3^-$	183.0117	[10]	√	√
84	6.15	Linoleic acid	279.2326	$C_{18}H_{31}O_2^-$	261.2199; 59.0138	[5]	√	√
85	6.32	Palmitic acid	255.2328	$C_{16}H_{31}O_2^-$	234.9956; 206.9872 166.9912	[19]	√	√
86	6.44	Oleic acid	281.2486	$C_{18}H_{33}O_2^-$	260.9945; 240.9874	[5]	√	√
87	6.74	Stearic acid	283.2632	$C_{18}H_{35}O_2^-$	242.9835;179.0094	[19]	√	√

√, found; -, not found; Al, *A. lucidior*; Ap, *A. procera*

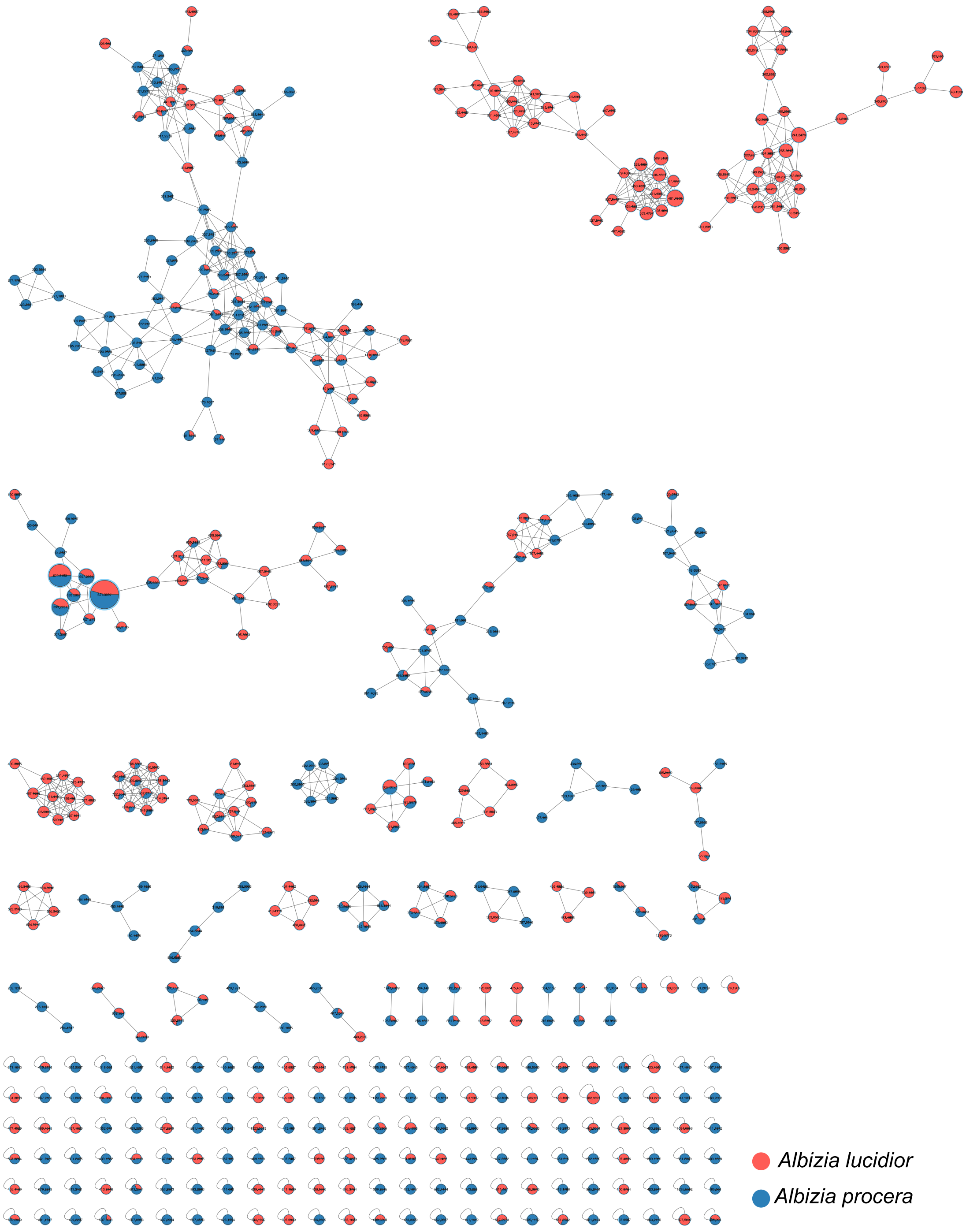

Figure S1. GNPS molecular network of two *Albizia* spp. ethanolic extracts in the positive ion mode. Each node is displayed as pie chart representing relative abundance of the metabolite with red and blue colors in the ethanolic extracts of *A. lucidior* and *A. procera*, respectively. The node label represents precursor mass (*m/z*). The node size represents the sum of precursor ion intensity.

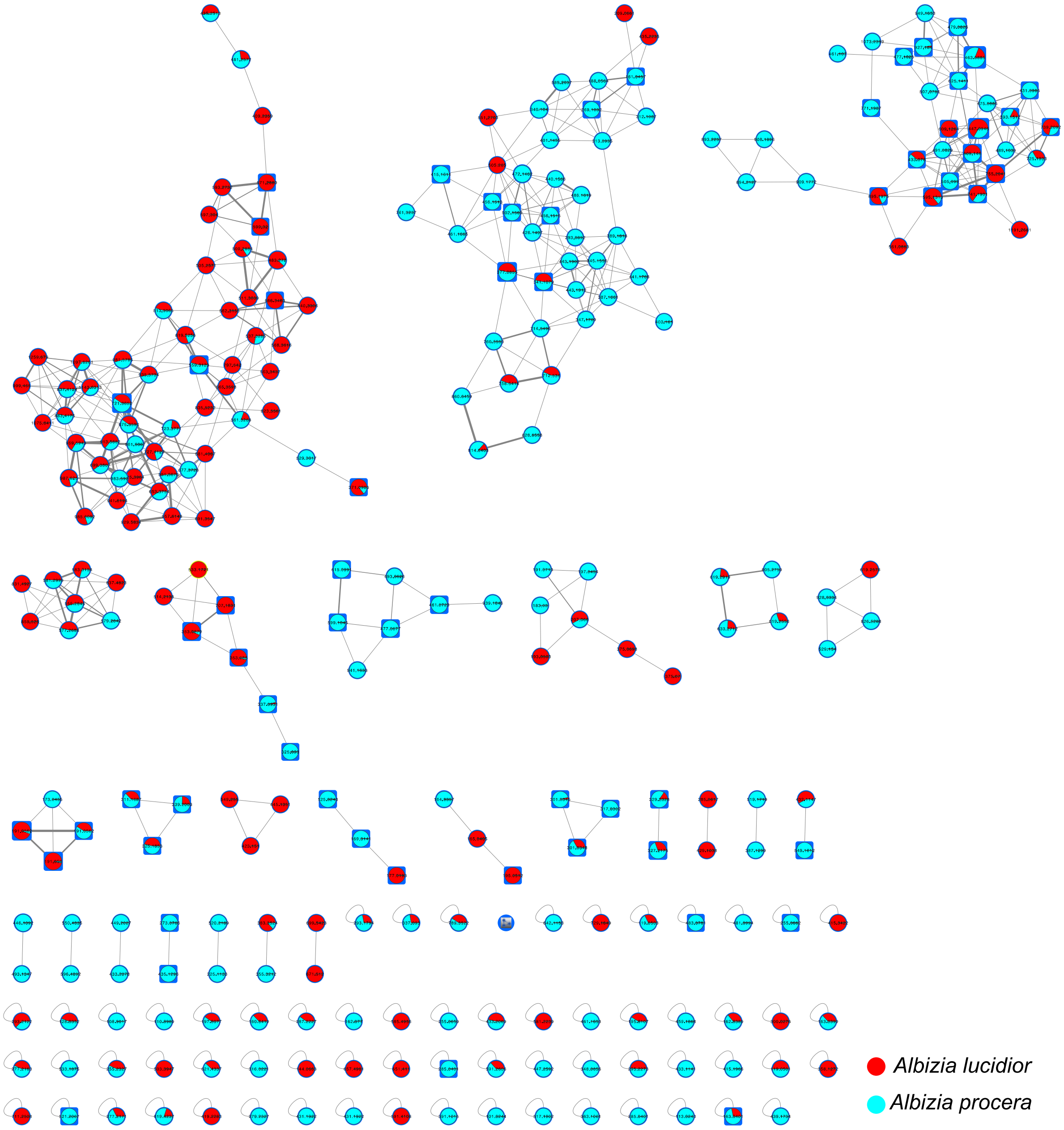

Figure S2. GNPS molecular network of two *Albizia* spp. ethanolic extracts in the negative ion mode. Each node is displayed as pie chart representing relative abundance of the metabolite with red and blue colors in the ethanolic extracts of *A. lucidior* and *A. procera*, respectively. The node label represents precursor mass (*m*/*z*). The node size represents the sum of precursor ion intensity.

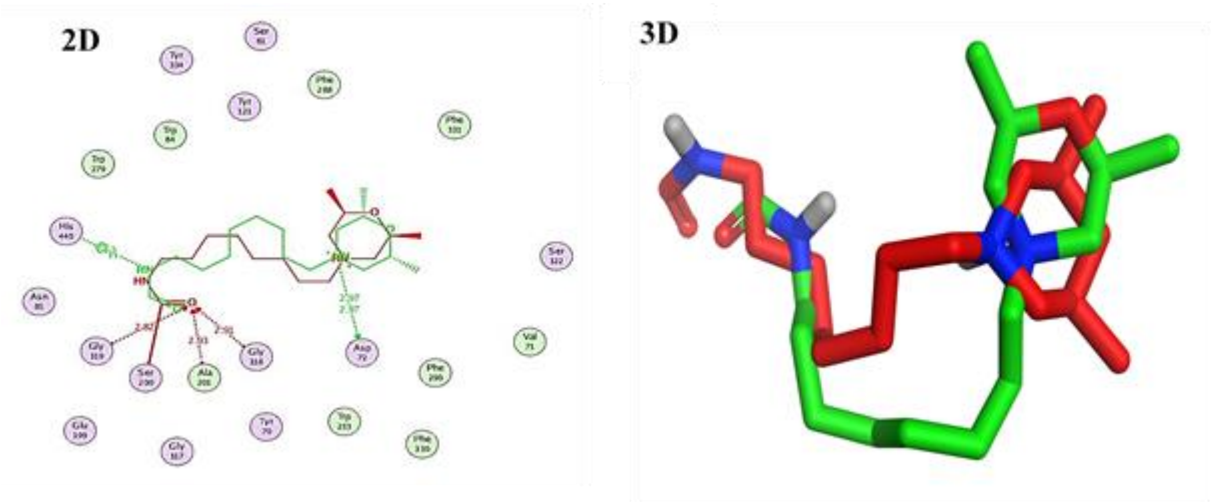

Figure S3. Superimposition of the redocked MF2 700 inhibitor (green) over its native one (red).

References

1. Kramberger, K.; Barlič-Maganja, D.; Bandelj, D.; Baruca Arbeiter, A.; Peeters, K.; Miklavčič Višnjevec, A.; Jenko Pražnikar, Z. HPLC-DAD-ESI-QTOF-MS determination of bioactive compounds and antioxidant activity comparison of the hydroalcoholic and water extracts from two *Helichrysum italicum* species. *Metabolites* **2020**, *10*, 403.
2. Rosa, W.; da Silva Domingos, O.; de Oliveira Salem, P.P.; Caldas, I.S.; Murgu, M.; Lago, J.H.G.; Sartorelli, P.; Dias, D.F.; Chagas-Paula, D.A.; Soares, M.G. *In vivo* anti-inflammatory activity of Fabaceae species extracts screened by a new *ex vivo* assay using human whole blood. *Phytochem. Anal.* **2021**, *32*, 859-883.
3. Singh, D.; Siew, Y.-Y.; Chong, T.-I.; Yew, H.-C.; Ho, S.S.-W.; Lim, C.S.E.-S.; Tan, W.-X.; Neo, S.-Y.; Koh, H.-L. Identification of phytoconstituents in *Leea indica* (Burm. F.) Merr. leaves by high performance liquid chromatography micro time-of-flight mass spectrometry. *Molecules* **2019**, *24*, 714.
4. Ateba, S.B.; Njamen, D.; Gatterer, C.; Scherzer, T.; Zehl, M.; Kählig, H.; Krenn, L. Rare phenolic structures found in the aerial parts of *Eriosema laurentii* De Wild. *Phytochemistry* **2016**, *128*, 5-11.
5. Kalogiouri, N.P.; Aalizadeh, R.; Dasenaki, M.E.; Thomaidis, N.S. Authentication of *Greek PDO kalamata* table olives: A novel non-target high resolution mass spectrometric approach. *Molecules* **2020**, *25*, 2919.
6. Shivanagoudra, S.R.; Perera, W.H.; Perez, J.L.; Athrey, G.; Sun, Y.; Wu, C.S.; Jayaprakasha, G.; Patil, B.S. *In vitro* and *in silico* elucidation of antidiabetic and anti-inflammatory

activities of bioactive compounds from *Momordica charantia* L. *Bioorg. Med. Chem.* **2019**, *27*, 3097-3109.
7. Sobeh, M.; Rezq, S.; Sabry, O.M.; Abdelfattah, M.A.O.; El Raey, M.A.; El-Kashak, W.A.; El-Shazly, A.M.; Mahmoud, M.F.; Wink, M. *Albizia anthelmintica*: HPLC-MS/MS profiling and *in vivo* anti-inflammatory, pain killing and antipyretic activities of its leaf extract. *Biomed. Pharmacother.* **2019**, *115*, 108882.
8. Mudrić, S.Ž.; Gašić, U.M.; Dramićanin, A.M.; Ćirić, I.Ž.; Milojković-Opsenica, D.M.; Popović-Đorđević, J.B.; Momirović, N.M.; Tešić, Ž.L. The polyphenolics and carbohydrates as indicators of botanical and geographical origin of Serbian autochthonous clones of red spice paprika. *Food Chem.* **2017**, *217*, 705-715.
9. Abu-Reidah, I.M.; Arráez-Román, D.; Warad, I.; Fernández-Gutiérrez, A.; Segura-Carretero, A. UHPLC/MS^2-based approach for the comprehensive metabolite profiling of bean (*Vicia faba* L.) by-products: A promising source of bioactive constituents. *Food Res. Int.* **2017**, *93*, 87-96.
10. Elkousy, R.H.; Said, Z.N.A.; Abd El-Baseer, M.A.; Abu El wafa, S.A. Antiviral activity of castor oil plant (*Ricinus communis*) leaf extracts. *J. Ethnopharmacol.* **2021**, *271*, 113878.
11. Elez Garofulić, I.; Malin, V.; Repajić, M.; Zorić, Z.; Pedisić, S.; Sterniša, M.; Smole Možina, S.; Dragović-Uzelac, V. Phenolic Profile, Antioxidant Capacity and Antimicrobial Activity of Nettle Leaves Extracts Obtained by Advanced Extraction Techniques. *Molecules* **2021**, *26*, 6153.
12. Sobeh, M.; Hassan, S.A.; El Raey, M.A.; Khalil, W.A.; Hassan, M.A.; Wink, M. Polyphenolics from *Albizia harveyi* exhibit antioxidant activities and counteract oxidative damage and ultra-structural changes of cryopreserved bull semen. *Molecules* **2017**, *22*, 1993.
13. Ruan, J.; Yan, J.; Zheng, D.; Sun, F.; Wang, J.; Han, L.; Zhang, Y.; Wang, T. Comprehensive chemical profiling in the ethanol extract of *Pluchea indica* aerial parts by liquid chromatography/mass spectrometry analysis of its silica gel column chromatography fractions. *Molecules* **2019**, *24*, 2784.
14. Fotirić Akšić, M.; Dabić Zagorac, D.; Sredojević, M.; Milivojević, J.; Gašić, U.; Meland, M.; Natić, M. Chemometric characterization of strawberries and blueberries according to their phenolic profile: Combined effect of cultivar and cultivation system. *Molecules* **2019**, *24*, 4310.
15. Limwachiranon, J.; Huang, H.; Li, L.; Duan, Z.; Luo, Z. Recovery of lotus (*Nelumbo nucifera Gaertn.*) seedpod flavonoids using polar macroporous resins: The updated understanding on adsorption/desorption mechanisms and the involved intermolecular attractions and bonding. *Food Chem.* **2019**, *299*, 125108.
16. Huang, Q.; Zhang, F.; Liu, S.; Jiang, Y.; Ouyang, D. Systematic investigation of the pharmacological mechanism for renal protection by the leaves of *Eucommia ulmoides* Oliver using UPLC-Q-TOF/MS combined with network pharmacology analysis. *Biomed. Pharmacother.* **2021**, *140*, 111735.
17. Chiriac, E.R.; Chiţescu, C.L.; Borda, D.; Lupoae, M.; Gird, C.E.; Geană, E.-I.; Blaga, G.-V.; Boscencu, R. Comparison of the polyphenolic profile of *Medicago sativa* L. and *Trifolium*

pratense L. sprouts in different germination stages using the UHPLC-Q exactive hybrid quadrupole orbitrap high-resolution mass spectrometry. *Molecules* **2020**, *25*, 2321.

18. Zhang, T.; Qiu, F.; Chen, L.; Liu, R.; Chang, M.; Wang, X. Identification and *in vitro* anti-inflammatory activity of different forms of phenolic compounds in *Camellia oleifera* oil. *Food Chem*.**2021**, *344*, 128660.
19. Farag, M.A.; Khattab, A.R.; Maamoun, A.A.; Kropf, M.; Heiss, A.G. UPLC-MS metabolome based classification of Lupinus and Lens seeds: A prospect for phyto-equivalency of its different accessions. *Food Res. Int.* **2019**, *115*, 379-392.
20. Song, Q.; Song, Y.; Zhang, N.; Li, J.; Jiang, Y.; Zhang, K.; Zhang, Q.; Tu, P. Potential of hyphenated ultra-high performance liquid chromatography-scheduled multiple reaction monitoring algorithm for large-scale quantitative analysis of traditional Chinese medicines. *RSC Adv.* **2015**, *5*, 57372-57382.
